# Knowledge, Attitudes, and Practices Regarding Analgesic Use Among Adults in the United Arab Emirates: A Cross-Sectional Study

**DOI:** 10.7759/cureus.91522

**Published:** 2025-09-03

**Authors:** Arwa Ahmed, Salma Eldesouki, Doa J Mirza, Omar Lootah, Ahmed Maalej, Donya Fathy, Maha Saber-Ayad

**Affiliations:** 1 General Practice, Al Qassimi Hospital, Sharjah, ARE; 2 Internal Medicine, Sheikh Tahnoon Bin Mohammed Medical City, Al Ain, ARE; 3 Emergency Medicine, Al Qassimi Hospital, Sharjah, ARE; 4 General Medicine, University of Sharjah, Sharjah, ARE; 5 Clinical Sciences, University of Sharjah, Sharjah, ARE

**Keywords:** analgesic, knowledge, over the counter, pain management, uae

## Abstract

Background

Analgesics are compounds capable of relieving pain without loss of consciousness and are among the most commonly used medications, available both by prescription and over the counter. Despite their recognized adverse effects, the prevalence of analgesic use is on the rise, highlighting the need to study the knowledge, attitudes, and practices related to their use among the adult population of the United Arab Emirates (UAE).

Aim

We aimed to evaluate the knowledge, attitudes, and practices related to analgesic use in the adult population of the UAE.

Subject and methods

A cross-sectional study was conducted between February and March 2021, targeting adults aged 18 and above in the UAE. A self-administered online questionnaire was distributed via social media platforms, with participation being strictly voluntary. The questionnaire comprised 37 questions divided into four sections: demographics, knowledge, attitudes, and practices related to analgesic use.

Results

Of the 385 participants, 48% (n=185) reported using analgesics. Among these 185 participants, 88.7% (n=164) obtained analgesics over the counter, with paracetamol being the most commonly used. Additionally, 77% (n=296) of the 385 participants displayed poor knowledge regarding the appropriate use of analgesic medications, while 55% of our participants (n=212) had positive attitudes towards the correct usage of analgesics. Significant associations were found between age and knowledge score, the prevalence of over-the-counter analgesic use, and knowledge about adverse effects (p<0.05).

Conclusion

Our study highlights significant analgesic consumption in the UAE, however there is evidence of poor knowledge and harmful practices related to its use. The adverse effects associated with analgesics may lead to severe complications; therefore, proper educational and awareness campaigns focusing on analgesic use must be conducted.

## Introduction

Analgesics are drugs capable of relieving pain without loss of consciousness and are among the most commonly used medications, available both by prescription and over-the-counter (OTC). These medications are commonly used worldwide and are categorized as opioid and non-opioid analgesics. Opioid analgesics, such as morphine and codeine, are typically reserved for severe or chronic pain and are prescribed under the supervision of a physician [1] due to their addictive properties. Non-opioid analgesics, such as paracetamol and nonsteroidal anti-inflammatory drugs (NSAIDs), are available both by prescription and OTC. Unlike opioid analgesics, NSAIDs are used for mild to moderate pain control. Pain is a leading cause for doctor visits worldwide and is a significant cause of morbidity, making analgesic medications among the most commonly sought medications [2]. In the UAE, acetaminophen, a non-opioid analgesic, is the most commonly used analgesic [3].

Analgesic consumption has been rising during recent decades owing to the prevalence of chronic pain, the aging population, and new treatments [4]. In the Middle East, particularly in countries like Saudi Arabia, where the population is similar to that of the UAE, there has been a progressive increase in the utilization of these self-administered medications [5]. Emerging infectious diseases such as the COVID-19 pandemic have also influenced the trend of analgesic consumption worldwide, contributing to the rising trend of both OTC non-opioid analgesics as well as prescribed opioid analgesics [6].

NSAIDs, which help relieve pain can cause multiple adverse effects such as gastrointestinal bleeding, renal disease, and cardiovascular complications if abused or taken for extended periods, which poses a public health concern [7]. Paracetamol is one of the most widely used pain relievers globally and is considered safe because it is sold as an OTC medication; however, it can cause severe liver injury due to overdose, long-term use, or interaction with alcohol [8]. Similarly, aspirin and ibuprofen, among other NSAIDs, are often taken regularly without awareness of the adverse effects they pose, including gastrointestinal bleeding and cardiovascular issues [9].

Non-opioid analgesics have also been associated with exacerbations of existing conditions, such as Crohn’s disease, where those who consumed NSAIDs frequently were at a greater risk of active disease during follow-up [10]. An association between the use of NSAIDs such as celecoxib and rofecoxib and the exacerbation of heart failure has also been found [11].

The knowledge, attitudes, and practices regarding these drugs vary significantly across different populations and regions, influenced by cultural differences, accessibility of health services, and health policies [12]. It is essential to understand the pattern of their use in the country. In the UAE, the accessibility of OTC analgesics and low awareness of the dangers of improper use raise concerns about the potential for overuse and the emergence of adverse health consequences [13].

OTC availability of most analgesics makes them easily accessible, alongside the cultural acceptability of self-administered drugs and the lack of strict regulations restricting the sale of certain non-prescription drugs in the UAE [14]. Furthermore, the population in the UAE consists of nationals and foreigners from diverse cultural backgrounds, implying that they have different attitudes and practices towards pain management. For instance, expatriates from developed Western countries might be more accustomed to the frequent prescription of analgesics. In contrast, individuals from cultures that are more in touch with traditional medicine are likely to use pharmacological means less frequently [15].

Understanding the appropriate timing and rationale for administering analgesics through physicians is crucial to minimizing misuse and reducing potential adverse consequences as well as reducing the risk of intentional medication abuse. In the UAE, this issue is exacerbated by the common belief that OTC medications like acetaminophen or ibuprofen are harmless since they can be purchased without a doctor’s prescription. This belief fosters a culture where people are willing to take analgesics even in situations where they may not need them or where it may be dangerous to do so [16]. This poses a significant concern due to a lack of knowledge regarding adverse effects and the concurrent use of medications that may interact with each other [17]. To illustrate this concern, [12] a study conducted in Saudi Arabia revealed that individuals take higher doses of an analgesic or for an extended period of time against medical advice. 

This study aims to measure the knowledge, attitudes, and practices regarding the utilization of non-opioid analgesics among adults in the UAE. Emphasis will be placed on aspects of self-medication, including patterns, risk awareness, and influencing factors related to the use of non-opioid analgesics to enhance safe pain management within the population. 

## Materials and methods

Study design and population

The required ethics approval was obtained from the University of Sharjah's ethics committee before the study was conducted (approval no. REC-21-02-10-05-S). It was a cross-sectional, descriptive study, and a convenience sampling method was used to collect data from adults residing in the UAE between February and March 2021. The questionnaire was distributed through social media platforms, like WhatsApp, Instagram, and Twitter. A participant information sheet was presented at the beginning of the questionnaire, where participants indicated their consent to participate in the study. The sample size of 385 was calculated for a population of 9.9 million, a 5% margin of error, and a 95% confidence interval. Individuals who could not speak English or Arabic, were under 18 years of age, or who used opioid analgesics were excluded from this study. 

Questionnaire development

Since there are no existing tools measuring the knowledge, attitudes, and practices of analgesic use, we developed one. The literature was reviewed, and similar studies were used as a guide to structure the questionnaire [16,18]. It comprised 39 questions and was divided into four sections: demographics, knowledge, attitudes, and practices. All questions were close ended, and in the form of single-choice and multiple-choice questions. The questionnaire was first developed in English and later translated to Arabic. Both versions were revised by a biostatistician and language specialists to ensure consistency. The questionnaire was then piloted and adjustments were done according to comments received. Participant responses were scored systematically. In the knowledge section, each correct answer on painkillers, their uses, and adverse effects was awarded one point, with a maximum score of 18. In the practices section, only participants who reported using painkillers were assessed, while non-users were excluded. In the attitudes section, agreement with statements was scored as two points, neutral as one point, and disagreement as zero points, yielding a maximum possible score of eight.

Data analysis

The responses obtained after the data collection process were exported to Microsoft Excel (Microsoft Corp., Redmond, WA, US), where data cleaning was performed, including the elimination of duplicate and irrelevant responses. A total of 422 valid responses were gathered. The cleaned dataset was then exported to the IBM SPSS Statistics for Windows, Version 26 (Released 2019; IBM Corp., Armonk, New York, United States) for further statistical analysis. Descriptive analysis was conducted to critically examine key variables, with outcomes described using frequency tables and percentages. The data was visualized using bar charts to display the knowledge, attitudes, and practices of the study participants regarding analgesic use. Bivariate analysis was done using the Kruskal-Wallis and Mann-Whitney U tests to study any significant associations between our variables, considering non-parametric tests for data that are not normally distributed. A cut off point of p-value <0.05 was used to denote significant difference between groups.

## Results

Demographics 

Out of the total number of respondents (n=422), those who consumed opioid analgesia were excluded. The demographic data for the rest (n=385) is given in Table 1.

**Table 1:** Demographics of the respondents on non-opioid analgesics (n=385)

Demographics	Categories	% (n)
Age (years)	18 - 24	53.6% (n=206)
	25 - 34	13.7% (n=53)
	35 - 44	15.4% (n=59)
	45 and above	17.3% (n=67)
Sex	Male	27% (n=104)
	Female	73% (n=281)
Nationality	Non-Arab	17.1% (n=66)
	UAE National	29.9% (n=115)
	Other Arab	53% (n=204)
Emirate	Abu Dhabi	24.4% (n=94)
	Dubai	32% (n=123)
	Ras Al-Khaimah	1.4% (n=5)
	Um el Quwain	0.9% (n=3)
	Ajman	8.8% (n=34)
	Fujairah	2.8% (n=11)
	Sharjah	29.6% (n=114)
Marital status	Single or otherwise	64.7% (n=249)
	Married	35.3% (n=136)
Highest degree	Secondary Education	24.4% (n=94)
	Diploma	8.8% (n= 34)
	Bachelor degree	52.2% (n=201)
	Higher education	14.5% (n=56)
Occupation	Unemployed	20.8% (n= 28)
	Student (non-healthcare major )	11.7% (n= 45)
	Student (healthcare major)	36.1% (n=139)
	Non healthcare worker	24.2% (n=93)
	Healthcare worker	7.3% (n=28)
Comorbidities	Hypertension	7.01% (n=27)
	Diabetes mellitus	6.23% (n=24)
	Hyperlipidemia	5.71% (n=22)
	Gastrointestinal disease	5.45% (n=21)
	Bone disease	3.37% (n=13)
	Cardiovascular/Respiratory diseases	2.85% (n=11)
	Kidney disease	1.03% (n=4)
	Others	2.33% (n=9)
Painkiller consumption	Yes	48.1% (n=185)
	No	51.9% (n=200)

The majority of our participants were aged between 18 and 24 years (53.6%), with 73% being female respondents, non-Emirati Arabs (53%), and single (64.7%). Around 52.2% had a Bachelor's degree, and 7.3% of them were healthcare sector employees. A greater proportion of our participants were not diagnosed with any type of chronic disease (66.02%), with about 48.1% consuming OTC and/or prescription analgesics. 

**Figure 1:**
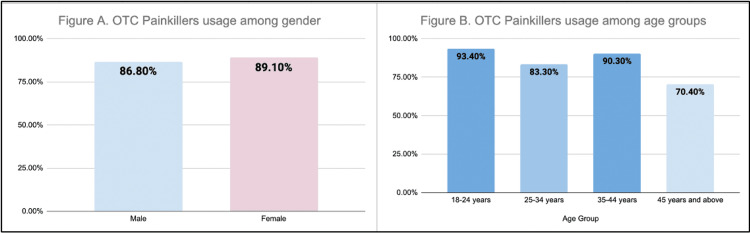
OTC painkiller usage stratified as per (A) gender and (B) age group (n=385)

Figure 1A shows the distribution of OTC painkiller use by gender.

Among male participants, 86.8% reported taking painkillers, which accounted for 16.3% of the total sample. Among female respondents, 89.1% reported using painkillers, representing 72.4% of the total sample. Figure 1B presents the use of OTC painkillers across different age groups. It demonstrates that OTC painkiller use is prevalent across all age groups, with a total of 88.7% (n=164) of participants reporting their use. In the youngest group (18-24 years), 93.4% of them reported taking OTC painkillers, making up 55.7% of the total sample.

Table 2 presents the cross-tabulation of age groups and the use of doctor-prescribed painkillers among participants.

**Table 2:** Responses to the query 'Do you take painkillers prescribed by a doctor?' arranged by age group (n=385)

Age groups	18-24 years	25-34 years	35-44 years	45 years and above	Total
Do you take painkillers prescribed by a doctor?					
Yes					
Count	39	10	14	16	79
% within "Do you take painkillers prescribed by a doctor?"	49.4%	12.7%	17.7%	20.3%	100.0%
% within Age groups	32.2%	41.7%	45.2%	59.3%	38.9%
% of Total	19.2%	4.9%	6.9%	7.9%	38.9%
No					
Count	82	14	17	11	124
% within "Do you take painkillers prescribed by a doctor?"	66.1%	11.3%	13.7%	8.9%	100.0%
% within Age groups	67.8%	58.3%	54.8%	40.7%	61.1%
% of Total	40.4%	6.9%	8.4%	5.4%	61.1%
Total					
Count	121	24	31	27	203
% within "Do you take painkillers prescribed by a doctor?"	59.6%	11.8%	15.3%	13.3%	100.0%
% within Age groups	100.0%	100.0%	100.0%	100.0%	100.0%
% of Total	59.6%	11.8%	15.3%	13.3%	100.0%

Overall, 38.9% of respondents reported using painkillers prescribed by a doctor, with the highest usage observed in the 45 years and above age group (59.3%). In contrast, only 32.2% of those aged 18-24 reported the use of doctor-prescribed painkillers, despite this group accounting for nearly half (49.4%) of all individuals who reported such use.

These findings suggest that older individuals are more likely to use doctor-prescribed painkillers, possibly reflecting greater healthcare needs or more frequent interactions with healthcare providers. Meanwhile, younger participants may be more inclined to self-medicate with non-prescription options.

**Figure 2:**
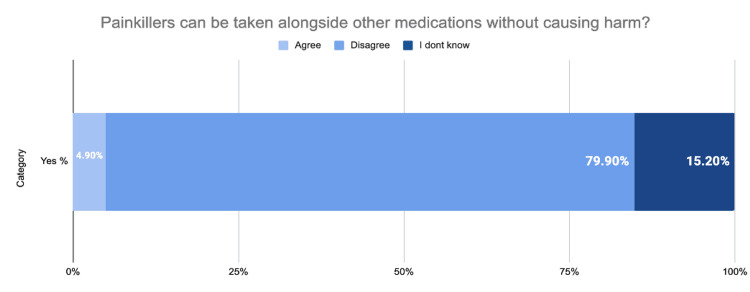
Knowledge about taking painkillers with other medications (n=385)

Figure 2 provides insights into the knowledge about taking painkillers with other medications among individuals with long-standing illnesses.

The table categorizes responses into three groups: those who agree, disagree, and those who are unsure about the statement that people with long-standing illnesses can take any type of painkillers.

The data revealed that a significant majority of respondents, 79.9% (n=308), disagreed with the statement, indicating a high level of awareness about the potential risks associated with taking painkillers indiscriminately with other medications. In contrast, only 20.1% (n=77) of respondents agreed with the statement. Overall, the data underscores a prevalent cautious attitude among individuals with long-standing illnesses regarding the concurrent use of painkillers and other medications.

As shown in Table 3, based on participants' age and educational level, the Kruskal-Wallis test findings revealed statistically significant variations in knowledge and experiences related to analgesics.

**Table 3:** Descriptive statistics for knowledge, attitude, and practice scores

Variable	Mean	Median	Standard deviation	Minimum
Attitude scores	5.51	6	1.538	0
Knowledge scores	6.72	12	2.275	0
Practice scores	Was not obtained			

Particularly, knowledge scores varied greatly between the age groups (p<0.05), implying that age is a major factor influencing people's awareness of analgesic use. The age-related variation in the occurrence and reporting of negative side effects linked to analgesics also suggested different experiences or practices between younger and older populations. Moreover, notable differences were seen in both the awareness of possible negative effects and general knowledge of analgesic use depending on educational level (p<0.05), emphasizing education as a major influencing element.

Additionally, the overall analysis derived from the knowledge and attitude scores (Table 3) revealed that 77% of participants (n=296) demonstrated poor knowledge regarding the appropriate use of analgesic medications, while 55% (n=212) had positive attitudes towards correct analgesic use. The poor knowledge levels are further supported by the low mean knowledge score of 6.72 (out of a maximum possible score of 18), indicating limited understanding among the majority of participants. These findings reinforce the need for targeted educational interventions. Collectively, the results highlight the importance of demographic factors-particularly age and level of education-in influencing knowledge, attitudes, and experience outcomes related to analgesic use.

## Discussion

Analgesics, commonly referred to as pain-relieving medications, are extensively utilized across the globe [19], with the market in the United Arab Emirates reaching a revenue of US $126.40 million in 2024 and projected to grow at an annual rate of 7.4%. However, the understanding, attitudes, and practices related to these drugs differ markedly among various populations and regions. These variations are shaped by cultural distinctions, the availability of healthcare services, and prevailing health policies [12]. Several studies have focused on the analgesic consumption in different populations. Our results are consistent with many of the previous reports, while also providing additional information in the context of the UAE. In a similar cross-sectional study conducted in the UAE, data were collected on the prevalence of OTC medication use among a sample of 6,363 adolescents. The study found that 51% of participants reported using OTC medications, with acetaminophen being the most commonly used form [3]. Similarly, our study revealed that 48.1% of participants consume analgesics, including OTC and prescribed ones. 

Our results also indicated that female participants used OTC analgesic drugs significantly more frequently than male respondents, with 81.7% of the former compared to 18.3% of the latter. This pattern aligns with several previous studies, which also found higher analgesic usage among the female population [20]. Another study [21] revealed that women often use OTC analgesics for headaches, back pain, and neuropathic pain. Additionally, conditions related to chronic pain, such as rheumatic disorders and osteoarthritis, are more prevalent among women [21]. Sex hormones affect pain perception differently in the male and female populations; estrogen is associated with both increased and decreased sensitivity to pain, whereas testosterone typically has a protective role [20]. Conversely, males may self-prescribe lower pain relief or may not report pain and seek treatment due to cultural biases. Research on behavioral sex/gender differences shows that women often adopt a wider variety of pain-coping strategies, frequently incorporating social support, and display greater engagement in healthcare-seeking behaviors such as using analgesics or visiting physicians. Conversely, men tend to rely more on avoidance or distraction and are less inclined to seek medical assistance [20]. These results raise concern towards the rising use of analgesics among the female population and warrant the need to find alternatives that are associated with similar efficacy and fewer adverse effects.

A systematic review concluded that certain formulations that have natural origins such as Zingiber montanum oil and Mchamomilla, contain analgesic and anti-inflammatory properties that contribute to improving blood circulation, reducing the inflammatory process, and other benefits, and can be useful in conditions such as osteoarthritis [22], essentially providing the same effect of analgesics without the increased risk of adverse effects. 

A recent study identified a high level of self-medication among UAE residents, with paracetamol and ibuprofen being the most commonly used OTC medications [23]. Similarly, our study found that the most frequently reported medications among participants were specifically paracetamol and NSAIDs. This trend may be attributed to the ease of access to these drugs without a prescription, the prevalent culture of self-medication, and a lack of awareness regarding the potential dangers of misusing pain-relieving medications.

The findings of the current study reveal significant differences in the extent and patterns of analgesic consumption across various age groups. Notably, younger age groups, specifically those aged 18-24 and 25-34, exhibit consumption patterns that are consistent with previous research [24,25]. The primary reasons contributing to higher self-medication among young adults include increased physical activity, a higher likelihood of accidental injuries, pain or symptom development, and better access to OTC products. Notably, 4% of participants aged 18-24 years have used OTC non-prescription pain relievers. This finding aligns with the higher usage patterns observed by Aboalrob et al. [26], which indicate that populations in the Middle East frequently use painkillers, particularly for complaints such as headaches, menstrual pain, and muscular aches. 

In our study, there were no significant differences in attitudes toward analgesic use among the participants of different ethnicities (p=0.754). This finding is somewhat surprising given the diverse demographics of the UAE population, which includes various cultural and healthcare beliefs. However, it may also indicate the influence of globalized media and healthcare information, which promote universal concepts about pain control and drug use.

These differences raise concerns about the inadequate knowledge coverage in certain groups, highlighting the need for targeted education (p=0.023). Similar to the findings of Al Essa et al. [16], there are significant gaps in the knowledge regarding safe analgesics within the population, particularly among young people and those with lower levels of formal education. These findings suggest that the current public health promotion and communication efforts must be enhanced to effectively address and meet the needs of all population sectors [16].

Given the widespread availability of OTC analgesics and the knowledge gaps identified in this study, it is essential to enhance the policy framework regulating the sale and use of these drugs in the UAE. Currently, these non opioid analgesics are easily accessible without a prescription, which can lead to their misuse and potential adverse health consequences. To mitigate this risk, it is recommended to impose higher restrictions on the quantity of analgesics that can be sold at one time, require pharmacists to provide information on their proper use, and ensure that labels clearly and prominently display potential side effects. These measures can potentially reduce misuse rates and promote safer use of analgesics.

The findings of this study enhance the current understanding of knowledge, attitudes, and practices regarding non-opioid analgesic use in the UAE, contributing valuable insights to the broader field of pain management and self-medication. However, our study has some limitations that should be noted. Firstly, the use of self-selection volunteer sampling raises concerns about external validity, which affects the generalizability of our findings to the broader population in the UAE. Other limitations include the potential risk of recall bias due to reliance on self reported data and the lack of back translation which could potentially result in misunderstandings. The exclusion of opioid users and non-English/Arabic speakers, due to ethical and logistical constraints, may limit generalizability by omitting subpopulations with potentially distinct analgesic use patterns. The questionnaire, although piloted on 10 participants to refine clarity and flow, has not undergone full psychometric validation, and such assessment is recommended for future research. In addition, the brief data collection period (February-March 2021) may not fully capture seasonal variations in analgesic use; however, by asking participants about typical monthly use and common reasons, we aimed to reflect habitual patterns. The conclusions drawn from this study are instrumental in addressing gaps in knowledge regarding the use of analgesics among the people of the UAE. Future research should employ random sampling methods to obtain a more representative sample.

## Conclusions

The findings of this research indicated a high percentage of OTC non-opioid analgesic use among residents of the UAE. Substantial deficiencies in knowledge were present, particularly among young people and those with limited formal education. These results emphasize the importance of targeted educational efforts. We propose targeted interventions, including the implementation of educational campaigns aimed at university students and manual laborers, as well as pharmacist-led counseling initiatives designed to enhance health literacy among patients with limited understanding. In addition, policy recommendations that advocate for restricting the quantity of OTC analgesics permitted per purchase and mandate prominently displayed warning labels on product packaging can improve consumer awareness and promote safer medication use. Future studies should include more diverse populations to enhance understanding and contribute to effective interventions. 

## Appendices

Questionnaire in English

*Demographics*

Do you currently live in the UAE? 1) Yes 2) No

1. To which age group do you belong? 1) Less than 18 years 2) 18-24 years 3) 25-34 years 4) 35-44 years 5) 45-55 years 6) Above 55 years

2. Do you consume opioid drugs (e.g. Heroin, Codeine, Morphine, etc.) as painkillers? 1) Yes 2) No

Please enter the FIRST 3 letters of your LAST name:

Please enter the LAST 3 digits of your phone number:

3. What is your gender? 1) Female 2) Male

4. Please specify your nationality:

5. Please check your highest level of education: 1) Primary education (Grade 1-6) 2) Secondary education (Grade 7-12) 3) Diploma 4) Bachelor’s degree 5) Higher education

6. Please choose your current marital status: 1) Single 2) Married 3) Divorced 4) Widowed 5) Other:

7. What is the emirate of your residence? 1) Abu Dhabi 2) Dubai 3) Sharjah 4) Ajman 5) Ras Al-Khaimah 6) Umm Al Quwain 7) Al Fujairah

8. What is your current occupation? 1) Healthcare worker (Doctor, Nurse, Dentist, Pharmacist, Healthcare Administration, etc.) 2) Non-Healthcare worker 3) Student (Medicine, Dentistry, Health Sciences, etc.) 4) Student (Non-healthcare major) 5) Housewife 6) Unemployed 7) Other:

9. Do you have a first-degree relative (parent, sibling or child) working in the healthcare industry? 1) Yes 2) No

10. Do you have any long-standing illnesses? 1) I do not have any long-standing illnesses 2) High blood pressure 3) High blood sugar 4) High cholesterol levels 5) Bone related diseases 6) Bleeding problems 7) Kidney problems 8) Stomach ulcers 9) Heart Diseases 10) Heartburn 11) Other:

11. How long have you been smoking? 1) I do not smoke 2) Less than a year 3) 1-3 years 4) 4-6 years 5) 7-10 years 6) More than 10 years

12. How often do you exercise? 1) I do not exercise 2) Once per week 3) 2-3 times a week 4) 4-5 times a week 5) 6-7 times a week

Practices

13. Do you take any painkillers at all (prescription or over the counter? 1) Yes 2) No

14. Do you take painkillers over the counter? 1) Yes 2) No

15. How often do you take painkillers over the counter? 1) Rarely 2) Sometimes 3) Often 4) Always

16. Which painkiller do you take over the counter the most? 1) Paracetamol/Adol (Paracetamol) 2) Voltaren/Cataflam (Diclophenac) 3) Advil/Brufen/Nurofen (Ibuprofen) 4) Aspirin 5) Aleve (Naproxen) 6) Other:

17. How many tablets of over the counter painkiller do you take at one time? 1) One 2) Two 3) Three 4) More than three

18. Please write the dose of one tablet of painkiller in milligram (mg):

19. How many days a week do you use over the counter painkillers? 1) 1 day 2) 2-4 days 3) 5-7 days 4) More than a week

20. Do you take painkillers prescribed by a doctor? 1) Yes 2) No

21. How often are you prescribed painkillers? 1) Rarely 2) Sometimes 3) Often 4) Always

22. Which painkiller where you prescribed the most? 1) Paracetamol/Adol (Paracetamol) 2) Voltaren/Cataflam (Diclophenac) 3) Advil/Brufen/Nurofen (Ibuprofen) 4) Aspirin 5) Aleve (Naproxen) 6) Other:

23. How do you use the painkillers prescribed by your doctor? 1) Continuously (Skip question 24) 2) Intermittently

24. For how many days were you prescribed the therapy? 1) 1 day 2) 2 days 3) 3 days 4) 4-7 days 5) More than 1 week

25. What do you take painkillers (prescription or over the counter) mainly for? 1) Headaches 2) Dental pain 3) Pain in joints 4) Muscular pain 5) Menstruation pain 6) Fever 7) Sore throat 8) Back pain 9) Other:

26. Do you take painkillers (prescription or over the counter) for other reasons? (pick all that applies) 1) I do not 2) Headaches 3) Dental pain 4) Pain in joints 5) Muscular pain 6) Menstruation pain 7) Fever 8) Sore throat 9) Back pain 10) Other:

27. How often do you require the use of painkillers for those reasons? 1) Once per month 2) 2-3 times per month 3) 4-5 times per month 4) More than 6 times per month

28. Do you read the instructions leaflet before you take any painkiller? 1) Yes 2) No

Knowledge

29. Which of the following side effects do you think painkillers can result in after long-term use? (pick all that applies) 1) Painkillers do not result in any long-term side effects 2) Kidney failure 3) Heart diseases like heart attack 4) Bleeding tendencies 5) Joint problems 6) Stomach ulcers 7) Liver problems 8) I don’t know 9) Other:

30. Which of the following painkiller do you think has the highest probability to result in serious side effects? 1) Paracetamol/Adol (Paracetamol) 2) Voltaren/Cataflam (Diclofenac) 3) Advil/Brufen/Nurofen (Ibuprofen) 4) Aspirin 5) Aleve (Naproxen) 6) I don’t know

Choose the correct answer for the following statements:

31. The higher the dose, the more harmful a painkiller is. 1) Agree 2) Disagree 3) I don't know

32. It is very important to read the instructions leaflet before using any painkiller.

1) Agree 2) Disagree 3) I don't know

33. The recommended dose of any painkiller differs from person to person.

1) Agree 2) Disagree 3) I don't know

34. Painkillers can be taken alongside other medications without causing any harm.

1) Agree 2) Disagree 3) I don't know

35. People with long-standing illnesses can take any type of painkillers. 1) Agree 2) Disagree 3) I don't know

Attitudes

Please rate the level of agreement with the following statements:

36. Painkillers should never be taken unless prescribed by a doctor. 1) Strongly disagree 2) Disagree 3) Neutral/I don't know 4) Agree 5) Strongly agree

37. The benefits of using painkillers outweigh the risks.

1) Strongly disagree 2) Disagree 3) Neutral/I don't know 4) Agree 5) Strongly agree

38. Painkillers can harm people since they are easily obtained without a medical prescription. 1) Strongly disagree 2) Disagree 3) Neutral/I don't know 4) Agree 5) Strongly agree

39. There are not enough educational or awareness programs regarding painkiller use, which may lead to misuse/abuse.

1) Strongly disagree 2) Disagree 3) Neutral/I don't know 4) Agree 5) Strongly agree

End of survey. Thank you for your response.
